# Metrabase: a cheminformatics and bioinformatics database for small molecule transporter data analysis and (Q)SAR modeling

**DOI:** 10.1186/s13321-015-0083-5

**Published:** 2015-06-23

**Authors:** Lora Mak, David Marcus, Andrew Howlett, Galina Yarova, Guus Duchateau, Werner Klaffke, Andreas Bender, Robert C Glen

**Affiliations:** The Centre for Molecular Informatics, Department of Chemistry, University of Cambridge, Lensfield Road, Cambridge, CB2 1EW UK; European Molecular Biology Laboratory - European Bioinformatics Institute (EMBL-EBI), Wellcome Trust Genome Campus, Hinxton, Cambridge, CB10 1SD UK; Unilever Research & Development, 40 Merritt Blvd, Trumbull, CT 06611 USA; Unilever Research & Development, Olivier van Noortlaan, 3133 AT Vlaardingen, The Netherlands; Haus der Technik e.V., Hollestrasse 1, 45127 Essen, Germany; Department of Surgery and Cancer, Faculty of Medicine, Imperial College London, SW7 2AZ London, UK

**Keywords:** Database, Substrate, Inhibitor, Inducer, Transport protein, Membrane protein, Metabolism, CYP, Drug transporter, Metabolite transporter

## Abstract

**Abstract:**

Both metabolism and transport are key elements defining the bioavailability and biological activity of molecules, i.e. their adverse and therapeutic effects. Structured and high quality experimental data stored in a suitable container, such as a relational database, facilitates easy computational processing and thus allows for high quality information/knowledge to be efficiently inferred by computational analyses. Our aim was to create a freely accessible database that would provide easy access to data describing interactions between proteins involved in transport and xenobiotic metabolism and their small molecule substrates and modulators. We present Metrabase, an integrated cheminformatics and bioinformatics resource containing curated data related to human transport and metabolism of chemical compounds. Its primary content includes over 11,500 interaction records involving nearly 3,500 small molecule substrates and modulators of transport proteins and, currently to a much smaller extent, cytochrome P450 enzymes. Data was manually extracted from the published literature and supplemented with data integrated from other available resources. Metrabase version 1.0 is freely available under a CC BY-SA 4.0 license at http://www-metrabase.ch.cam.ac.uk.

**Graphical Abstract:**

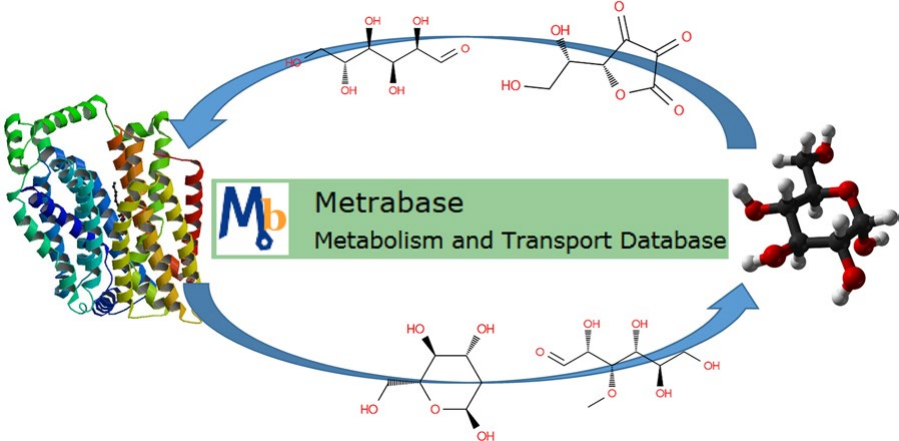

**Electronic supplementary material:**

The online version of this article (doi:10.1186/s13321-015-0083-5) contains supplementary material, which is available to authorized users.

## Background

Absorption, distribution, metabolism and excretion (ADME) properties of small molecules are vitally important for their function and potential toxicity, hence understanding their interaction with transport proteins as well as metabolizing enzymes is fundamental to the discovery and development of e.g. safe, efficacious medicines and skin products. However, this data is largely dispersed across the literature, with fractions of the data available in current open and proprietary databases. Numerous valuable publicly available transporter-related data sources exist that are either specific to membrane transporters (e.g. TP-search [1], UCSF-FDA TransPortal [2], TSdb [3], Human Transporter Database [4], HMTD [5], TCDB [6], SLC Tables [7] and Membrane Proteins of Known 3D Structure [8]) or contain transporter data as part of a broader collection of biological and pharmacological data (e.g. ChEMBL [9], HMDB [10], DrugBank [11], Transformer [12], KEGG [13], Recon X [14], PharmGKB [15], UniProt [16], CTD [17] and TTD [18]), but most of these databases do not include the additional related data (e.g. tissue expression) and metadata required for in-depth cheminformatics analyses. Ligand-based studies, however, often remain the main or only option for modeling transport proteins as targets, since there are not many 3D structures of small molecule transporters available, especially for human proteins. Moreover, since these are very flexible proteins [19–21], analyses involving the dynamics of the multiple transporter systems is often complex and often beyond the scope of presently available simulation capabilities. Nonetheless, quick and convenient access to chemical structures is often missing in these resources and sometimes only chemical names are provided, e.g. UCSF-FDA TransPortal (focused on FDA-approved drugs) and Transformer (focused on biotransformations of xenobiotics). In QSAR modeling and other cheminformatics applications however, molecular properties are typically calculated using 1D, 2D or 3D representations of molecular structures and thus access to accurate molecular structures is required. Tools that convert chemical names into structures (e.g. NCI/CADD Chemical Identifier Resolver [22] or OPSIN [23]) can be used, but due to the complexity of chemical naming they may fail or derive incorrect/inconsistent structures, hence thorough manual checking of structures is highly beneficial. Those few relevant freely and easily accessible resources that do provide chemical structures for download, such as ChEMBL, HMDB and Drugbank, all have different aims and contents (collections of bioactivity data, metabolite data and drug data, respectively). Furthermore, their transporter substrate-related content and transporter specific metadata is in general limited. In a recent study that is most related to the current work, Sedykh et al. [24] collected and published a significant amount of data pertaining to substrates and modulators of transport proteins with the aim of building predictive models for several transporters. Our efforts overlap, extend and complement theirs. In a single resource, which is not limited to drugs and only positive results, we include cheminformatics-specific data and functionality and also protein tissue expression levels and transporter locations in tissues that are often missing in other comparable databases.

Here we describe Metrabase, in which we offer easily accessible data on small molecule transport and metabolism, publicly available and useful for computational analyses and modeling.

Both metabolism and transport are key elements defining the bioavailability and biological activity of molecules, i.e. their adverse and therapeutic effects. Membrane transport proteins are a large and diverse group of proteins that are responsible for transporting a very diverse collection of molecules, including ions and small molecules across biological membranes. This can be observed in a relatively low-throughput fashion (transporters or carriers, including ATP-powered pumps) and also in a high-throughput fashion, e.g. up to 10^8^ water molecules or ions per second (channels) [25]. Metrabase includes a selection of efflux and influx transporters from the ABC [26] and SLC [7] families of transporters. They have attracted significant interest in recent years [27] and are also included in the revised guidance of the US Food and Drug Administration (FDA) and the European Medicines Agency (EMA) [28] for the approval of drugs. In the area of metabolism, Metrabase contains a limited set of the cytochrome P450 isozymes (CYPs) that have been selected as a major group of xenobiotic metabolizing enzymes [29]. Since drug absorption and excretion are influenced by both transport and metabolism, transporters and drug metabolizing enzymes can serve as a coordinated export system for toxic compounds and drugs, i.e. as a protective mechanism. Metrabase aims to provide comprehensive structural, physicochemical and biological data that can be used to infer the relationships between these transporters/enzymes and their ligands and its first release is presented here.

## Construction and content

**Figure 1:**
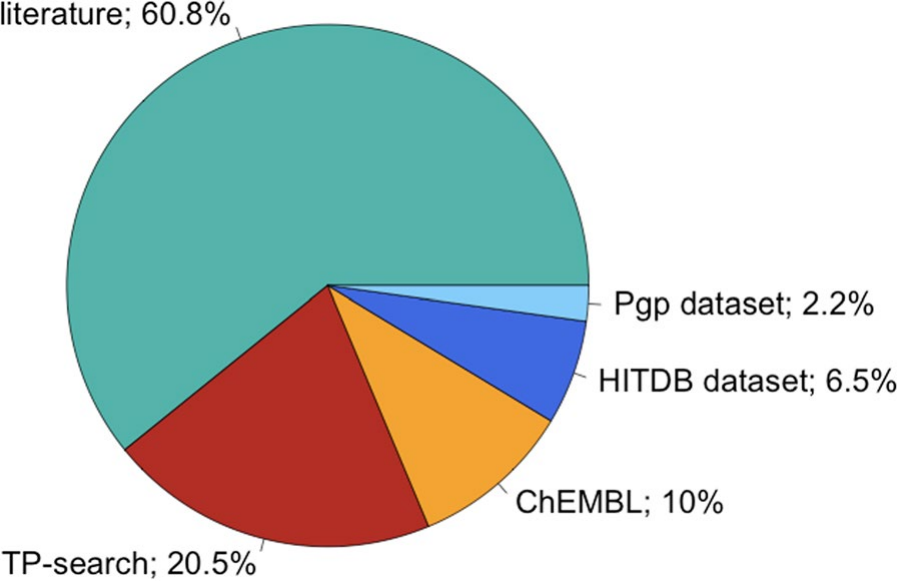
Fractions of Metrabase interaction records per data source. The total number of records is 11,649. ‘Pgp dataset’ and ‘HITDB dataset’ refer to the data taken from Poongavanam et al. [31] and Sedykh et al. [24] respectively. All the interaction data contained in Metrabase was extracted from the published literature: where datasource is ‘literature’, the data was extracted by members of the Metrabase Development Team.

Metrabase v1.0 includes interaction data on 20 transporters and 13 CYPs. However, the major focus of this first version of the database is on transport proteins: specifically, on their interactions with small molecules that were experimentally found to be (or not to be) substrates. Data held in Metrabase was manually extracted from the published literature and supplemented with data integrated from other available resources (Figure 1). The literature search, carried out using resources and tools such as PubMed, SciFinder, Web of Knowledge, Google, Google Scholar and iHOP [30], primarily involved scanning for reports of transporter (non-)substrates. Protein synonyms given in the ‘protein_synonyms’ database table were used to ensure a comprehensive coverage of the search space where needed. If the search returned publications describing action types other than substrate or non-substrate, the data was or was not extracted at the curator’s discretion. Manual data extraction was carried out by thirteen supervised undergraduate second and third year Natural Sciences students of the University of Cambridge (with specialization in chemistry and biochemistry). Each curator underwent induction including reading background reviews on transporters and metabolism and an explanation of the literature review process. The curation process involved reading scientific articles (abstracts and full texts) to ensure that the correct action type was assigned to the interacting small molecule and then transferring the data of interest into an electronic form. Two curators were involved in data extraction and verification from a single publication, each person performing one or the other task, but not both tasks for the same record. Special attention was given to the accuracy of chemical structures: three curators verified a single structure. Protein, compound, action type, publication and organism (to which the protein belongs) were extracted as a minimum, while the additional data (as described below) were extracted at a curator’s discretion if the data was available in the publication.

**Figure 2:**
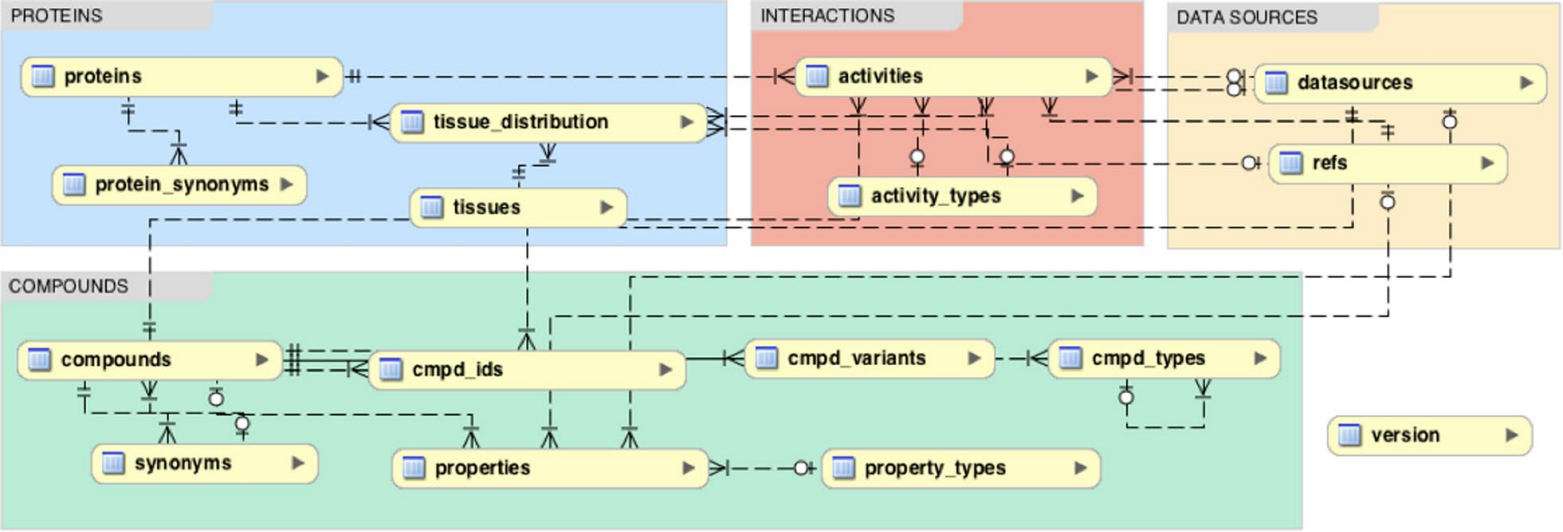
Metrabase schema. The database tables are grouped according to their context. There are four main tables: ‘activities’, ‘compounds’, ‘proteins’ and ‘refs’. Chemical structures and proteins are stored in the ‘compounds’ and ‘proteins’ tables respectively. Interactions between chemical compounds and proteins are stored in the ‘activities’ table, while the ‘refs’ table contains the publications’ citation information (bibliographic fields). An entity relationship diagram showing all the database fields is provided for download along with the database.

A summary of the transporter-related Metrabase content is shown in Table 1. The principal content is associated with 13 of 20 transporters (MDR1, BCRP1, PEPT1, MRP1-4, OCT1, OATP1B1, OATP2B1, OATP1B3, OATP1A2 and ASBT), while the remaining 7 transporters (OATP3A1, OATP4A1, MCT1, OATP2A1, OSTα/OSTβ, GLUT1 and LAT1) have many fewer associated records (less than 20 substrates/non-substrates). This initial selection of transporters was mainly directed by the guidelines of the International Transporter Consortium [27]—membrane transporters considered to have a role in drug absorption and disposition (distribution and elimination), therapeutic efficacy and safety (i.e. toxicity) were included. The total number of transporter-related activity records is substantial, totaling 11,143 records. The CYP-related content of Metrabase v1.0 is currently, however, limited to only 506 records for all the 13 CYP isoenzymes (3A4, 2S1, 2W1, 4B1, 2D6, 2C19, 2C9, 2E1, 1A2, 1A1, 1B1, 2C8 and 3A5). This selection of CYPs includes the major drug metabolizing isoforms (about 95% of the drugs are affected by them) and the isoforms expressed in the skin. The latter category includes mainly CYPs for which there is very little relevant experimental data published. The total numbers of compounds interacting with the 20 transporters and 13 CYPs are 3,307 and 212 respectively; these were compiled from 1,211 publications. On the biological side, Metrabase v1.0 also includes 1,087 records of transporter tissue expression levels manually extracted from 66 publications. We include the expression data, because the expression levels influence the capacity of transporters under different physiological and pathophysiological conditions. If, for example, we want to increase drug absorption using a transporter, then not only its substrate specificity, but also expression profile needs to be considered. In drug development, transporters expressed in the intestine, liver, kidney and blood–brain barrier are of particular interest. The overall database structure is shown in Figure 2.

**Table 1:** A summary of the transporter-related Metrabase content

Gene	Protein	Sub	nSub	−Mod	nInh	+Mod	nInd
ABCB1	MDR1	566	469	311	113	43	18
ABCG2	BCRP1	310	197	616	24	15	2
SLC15A1	PEPT1	247	95	275	26	7	–
ABCC2	MRP2	160	139	170	150	102	25
SLC22A1	OCT1	166	95	293	68	3	–
ABCC1	MRP1	98	91	4	4	–	–
SLCO1B1	OATP1B1	97	41	341	50	5	1
SLCO2B1	OATP2B1	48	75	136	203	23	–
SLCO1B3	OATP1B3	59	36	260	55	10	–
ABCC3	MRP3	66	24	47	4	17	5
SLCO1A2	OATP1A2	56	24	50	1	1	–
SLC10A2	ASBT	54	19	11	–	–	–
ABCC4	MRP4	47	19	–	–	–	–
SLCO3A1	OATP3A1	5	7	6	–	–	–
SLC16A1	MCT1	8	3	30	1	–	–
SLCO4A1	OATP4A1	7	1	4	–	–	–
SLC2A1	GLUT1	5	1	21	41	–	–
SLCO2A1	OATP2A1	5	1	9	3	–	–
SLC51A/B	OSTα/β	4	2	–	–	–	–
SLC7A5	LAT1	2	–	6	–	–	–

The figures quoted show the number of included compounds for their corresponding actions. Sub, nSub, **−**Mod, nInh, +Mod and nInd refer to substrate, non-substrate, negative modulators (inhibitor and repressor), non-inhibitor, positive modulators (stimulator and inducer) and non-inducer respectively.

### Activities

The key information held in the ‘activities’ table of the database covers the interactions between proteins and chemical compounds, indicating the compound action type as either substrate, non-substrate, inducer, non-inducer, repressor, inhibitor, non-inhibitor, stimulator or binder. In this work we define them as follows. Substrates are substances that are transported (by transporters) or catalyzed (by enzymes), while non-substrates are substances that were experimentally tested and found not to be substrates of a particular transporter or enzyme. Modulators (inducers, stimulators, inhibitors and repressors) are substances that alter the ability of a transporter to transport a substrate or alter the ability of an enzyme to metabolize a substrate. Inducers, non-inducers and repressors (associated with increased, unchanged and decreased levels of expression, respectively) belong to the compound action types related to protein expression, while inhibitors, non-inhibitors and stimulators (decreased, unchanged and increased protein activity, respectively) belong to the group of action types related to protein activity (Table 2). Care must be taken with respect to the current status of the inhibition records, since depending on the threshold used (e.g. percentage inhibition) some of the compounds annotated as ‘inhibitors’ can be regarded as ‘non-inhibitors’ and vice versa. This is an active area of research and is planned to be further resolved in subsequent releases of Metrabase.

**Table 2:** A list of the action types in Metrabase

	Action types
Protein activity (transport or catalysis)	Substrate Non-substrate
Compound activity (affecting protein activity/expression)	Inhibitor/repressor (negative modulators) Stimulator/inducer (positive modulators) Non-inhibitor/non-inducer (inactive compounds)

The ‘activities’ table was initialized with data taken from the TP-Search [1] and ChEMBL [9] databases, comprising 2,388 TP-Search records and 1,167 records holding action type annotations in ChEMBL versions 12 and 14. We also integrated the relevant data (1,009 records) from the recent publications of Sedykh et al. [24] and Poongavanam et al. [31]. Every interaction record is linked to the publication that provided the relevant data point. The current release holds only human-related records, but the importance of data related to orthologs is indisputable and therefore scheduled to be included in subsequent releases. There are five key activity fields: cmpd_id, protein_id, action_type, ref_id and species. Other ‘activities’ fields holding additional extracted data, such as assay descriptions, relevant experimental measurements, cell lines, compound concentrations and the substrates used in inhibition assays, may have only been partially completed in this release.

### Compounds

The total number of compounds with recorded interaction data for both transporters and enzymes is 3,438. Their structures are available in MDL molfile format and as absolute SMILES strings (in Kekulé form). The standard InChI and InChI Key strings were computed using v1.04 of the InChI software [32]. The great majority of the compounds are small organic molecules (containing just the following atoms: C, H, O, P, S, N, F, Cl, Br and I) and other types (coordination complexes, inorganic compounds, metalloid-containing compounds, selenium-containing compounds and polymers) are listed in the ‘cmpd_types’ table. Annotations of compounds as other types and subtypes, such as ‘natural product’ and ‘sesquiterpene’, can also easily be added. Stereoisomers, different forms (e.g. a cyclic isomer of glucose) and multi-component structures (e.g. irinotecan hydrochloride; also including mixtures, such as ivermectin) are listed in the ‘cmpd_variants’ table. Where it was not clear which component(s) of a multi-component structure are responsible for the action, the multi-component structure was annotated as a mixture (here only the mixture has been tested and so the biological activity may reside in either one or all of its individual components). Chemical structures were verified using ChemSpider [33] and SciFinder [34] as “gold standards”. The ‘properties’ table contains selected molecular properties, which were calculated/predicted for all structures (molecular mass) or just the small organic single-component structures (a selection of the constitutional descriptors, log *P* and log *D*) using ChemAxon’s Calculator (*cxcalc*) [35]. Additional properties, either calculated/predicted or experimental, can easily be added (and defined in the ‘property_types’ table before the records can be inserted). The database is therefore also expandable by other users who may wish to insert their own data or add metadata to the records.

### Proteins

The proteins contained in Metrabase are categorized as either transporters or enzymes and are provided with symbols and names approved by the HUGO Gene Nomenclature Committee (HGNC) [36], as well as UniProt [16] accession identifiers. Protein sequences for the indicated isoforms were included from UniProt. Metrabase also contains qualitative information about protein expression levels across healthy human tissues. Part of this data is based on immunohistochemistry using tissue microarrays and comes from the *normal_tissue.csv* file of the Human Protein Atlas (HPA) v9.0 [37]. All other expression records contain data extracted from the literature.

## Utility and discussion

### Database access

**Figure 3:**
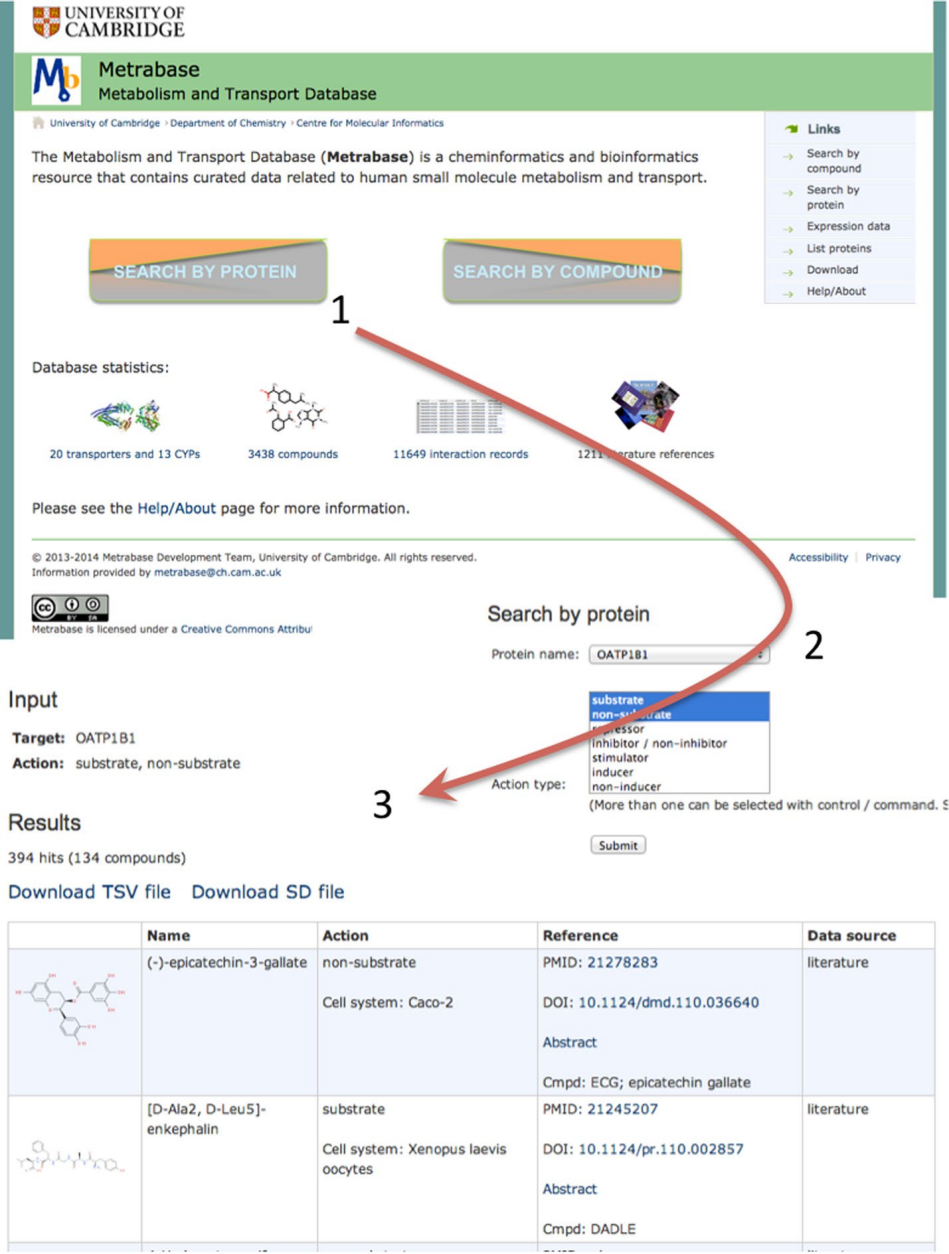
Search by protein example: retrieval of compounds tested for transport by OATP1B1. The Metrabase homepage is shown at the *top*. Results of searches are displayed in tables and can be downloaded in TSV (tab-separated values) and SD files.

Metrabase is accessible via an online user interface at http://www-metrabase.ch.cam.ac.uk, and allows users to search the database by protein or compound. Search by protein is the main feature (Figure 3). It allows retrieval of all compounds interacting with the selected protein and shows their action types and links to the literature references from which the data originated. Search by compound allows name, structure, substructure and similarity searches. These are supported by the ChemDoodle 2D sketcher [38] for structure drawing and the Mychem cartridge (for MySQL) [39], which performs cheminformatics-specific functions. The similarity search employs the FP2 fingerprint of Open Babel [40] and the exact search compares InChI strings. Furthermore, users can retrieve tissue expression data and access information held in the UniProt and HGNC databases following the links provided in the list of the proteins. A database dump is also available for download enabling users to install their own local copy of the database. MySQL Workbench can be used as an interface: even though knowledge of SQL may not be required to display records of all tables and save them in other formats, such as TSV or CSV, it is needed to fully exploit the database in this manner.

### The transporter substrate dataset

**Figure 4:**
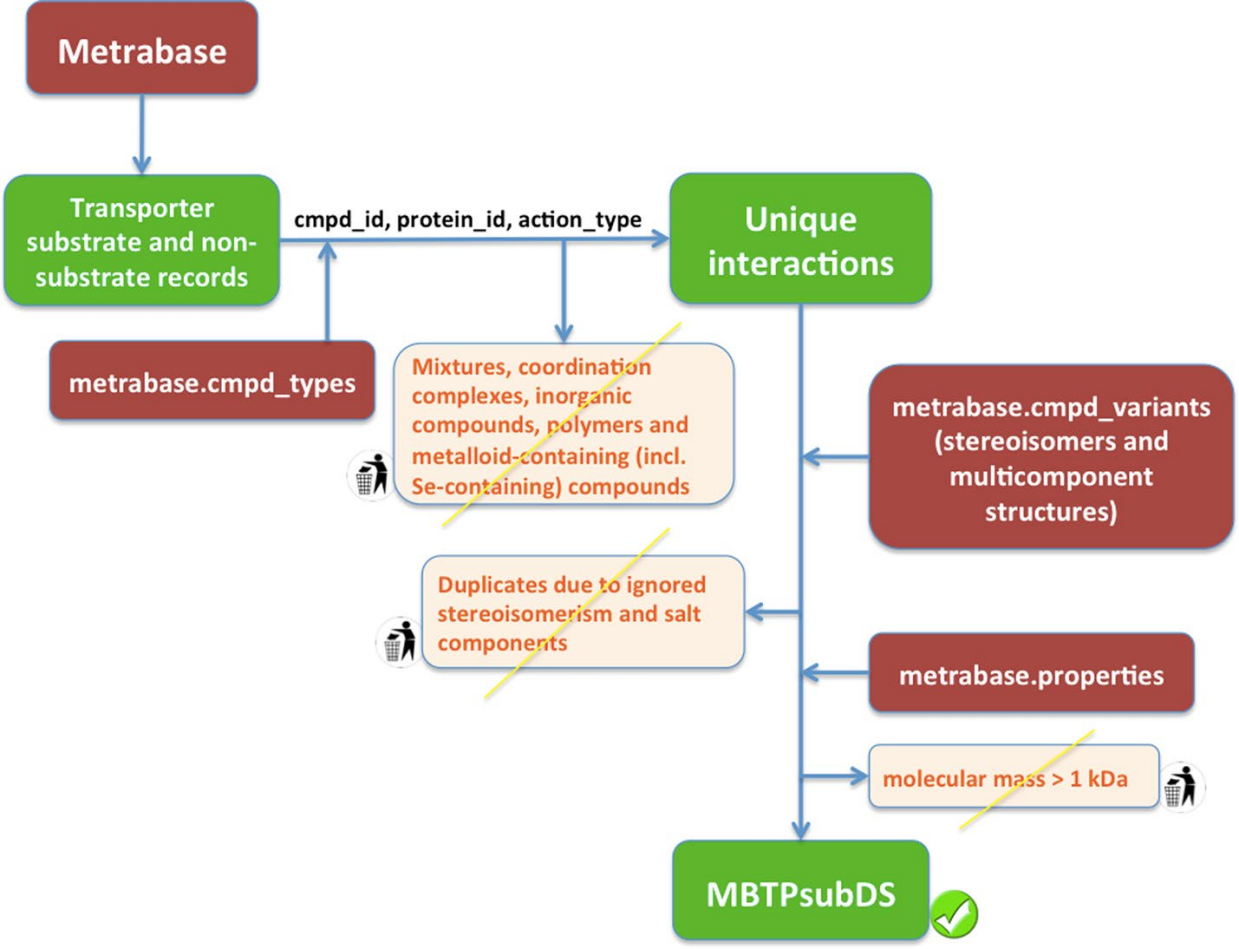
A diagram depicting the steps in the creation of the transporter substrate dataset. The first version of MBTPsubDS was generated using an in-house Python script to process the data retrieved from Metrabase. Mixtures, metal-, metalloid- and selenium- containing compounds and polymers were identified using the metrabase.cmpd_types table and compounds with molecular mass >1 kDa were identified using the metrabase.properties table.

We aim to provide a version of the transporter substrate dataset (MBTPsubDS) as a supplement to each Metrabase release (Figure 4). The first version of MBTPsubDS, based on Metrabase v1.0 and including datasets MBTPsubDS1_0 (Additional file 1) and MBTPsubDS1_0a (Additional file 2), was created using an in-house Python script and contains unique substrate and non-substrate records, which were processed to facilitate human transporter data analysis and predictive modeling. Mixtures, coordination complexes and other metal-containing compounds, inorganic compounds, polymers and metalloid-containing (including selenium-containing) compounds (as annotated in the ‘cmpd_types’ table) were removed. Only small molecules defined as those with a molecular mass of ≤1 kDa were retained. Stereoisomers were not considered (of course, this is an area for future development as stereoisomers can have substantially different bioactivities) and all the remaining multi-component structures were replaced by their single-component counterparts as given in the ‘cmpd_variants’ table. Effectively, this corresponds to the usually applied procedure of keeping the largest component of each structure, except that this procedure is based on either the number of atoms or molecular mass and can thus lead to retention of unwanted components. Finally, all the duplicates formed due to ignored stereoisomerism and salt components were removed.

**Figure 5:**
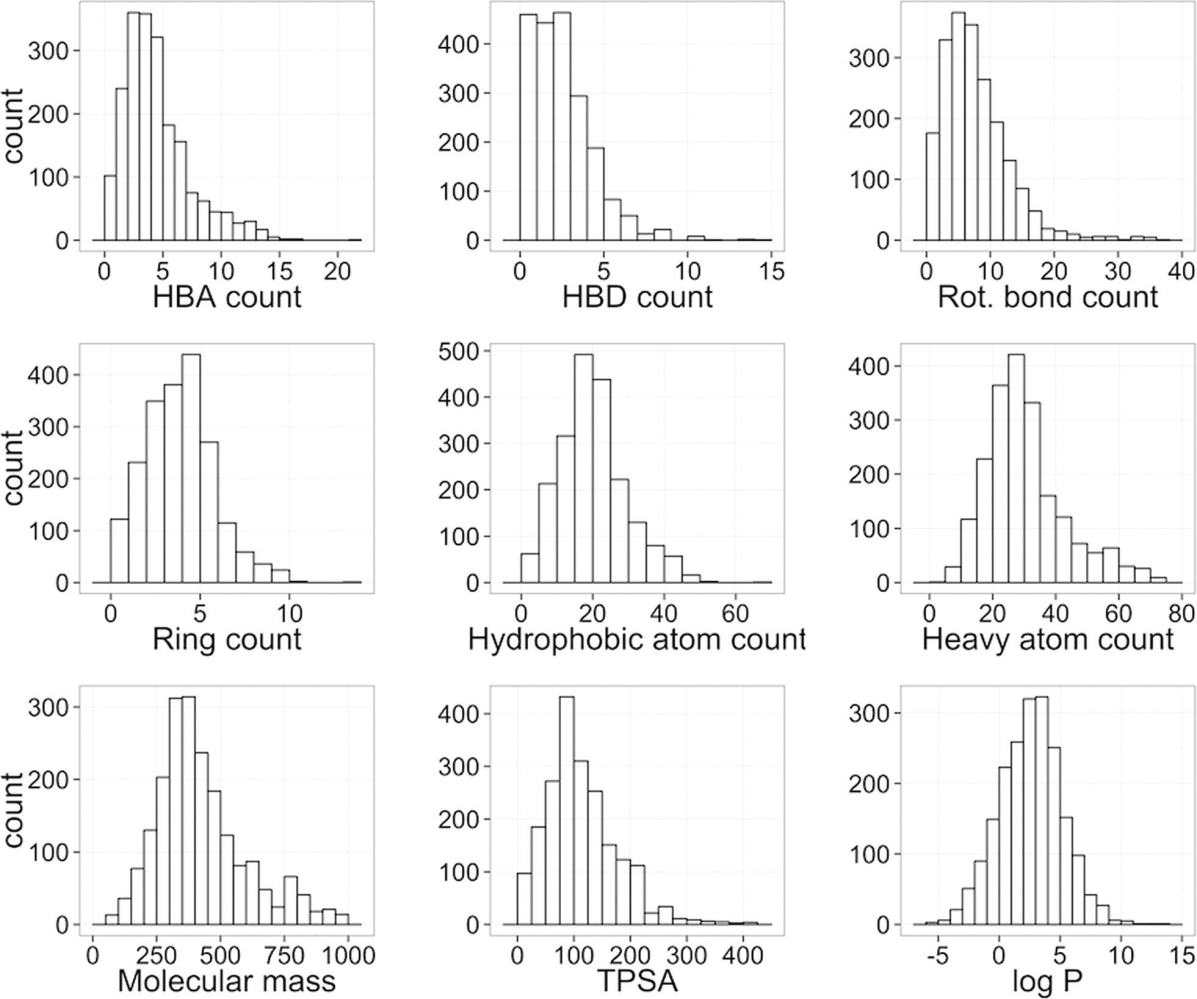
Histograms showing distributions of selected properties for all compounds contained in the MBTPsubDS1_0a dataset. HBA, HBD and TPSA stand for hydrogen bond acceptor, hydrogen bond donor and topological polar surface area respectively.

Datasets MBTPsubDS1_0 and MBTPsubDS1_0a contain 2,901 and 2,913 small molecule-transporter interaction records respectively. The latter dataset is a slightly extended version of the former dataset. All the interactions involving conflicting action types (where a compound was found to be both a substrate and a non-substrate of a single transporter) were removed from the former dataset, whereas the latter dataset contains a few of the compound-protein pairs corresponding to the resolved conflicting action types (where upon inspection we have defaulted to considering the compound as either a substrate or a non-substrate depending on the number of publications supporting each type). Compounds of the MBTPsubDS dataset make a diverse collection of molecular structures covering wide ranges of property values, however they are mostly lipophilic compounds, with 85% of the compounds possessing calculated log *P* > 0. Distributions of log *P* and several other selected molecular properties are shown in Figure 5.

### Usage

Structured and high quality experimental data stored in a container, such as a relational database, can facilitate easy computational processing, thus allowing for high quality information/knowledge to be inferred by computational analyses. In cheminformatics and other disciplines dealing with small molecules, in particular the correctness of chemical structures is vital [41, 42]. As stated by Young et al. [41], small structural errors can lead to significant errors in predictions and so the predictivity of QSAR models could be substantially increased by thorough curation.

Data mining using resources like Metrabase can be employed to understand, for example, transporter function or the promiscuous nature of compounds binding to transporters competitively or non-competitively. Transporters can be highly promiscuous proteins transporting structurally quite diverse compounds across cell membranes at rates of hundreds to tens of thousands per second [25]. We can infer the relationships between transporters and their ligands, to see what kind of substrates they prefer and do not prefer, and why. The intended purpose of the database is also to facilitate in silico predictive modeling, e.g. tissue distribution of a new compound (and thus its toxicity potential assessed within the scope of the predicted tissue accumulation).

MBTPsubDS1_0 and MBTPsubDS1_0a datasets were created using the data contained in Metrabase v1.0 to allow a structural analysis of the transporter substrates and generation of transporter models that can predict whether or not a compound is a substrate of a range of efflux and uptake transporters (this work will be published elsewhere). All the required information (e.g. stereoisomers, metal complexes or multi-component structures linked to their single-component counterparts) is held in the database, and thus only a processing script written in a suitable programming language is sufficient to produce a version of MBTPsubDS.

**Figure 6:**
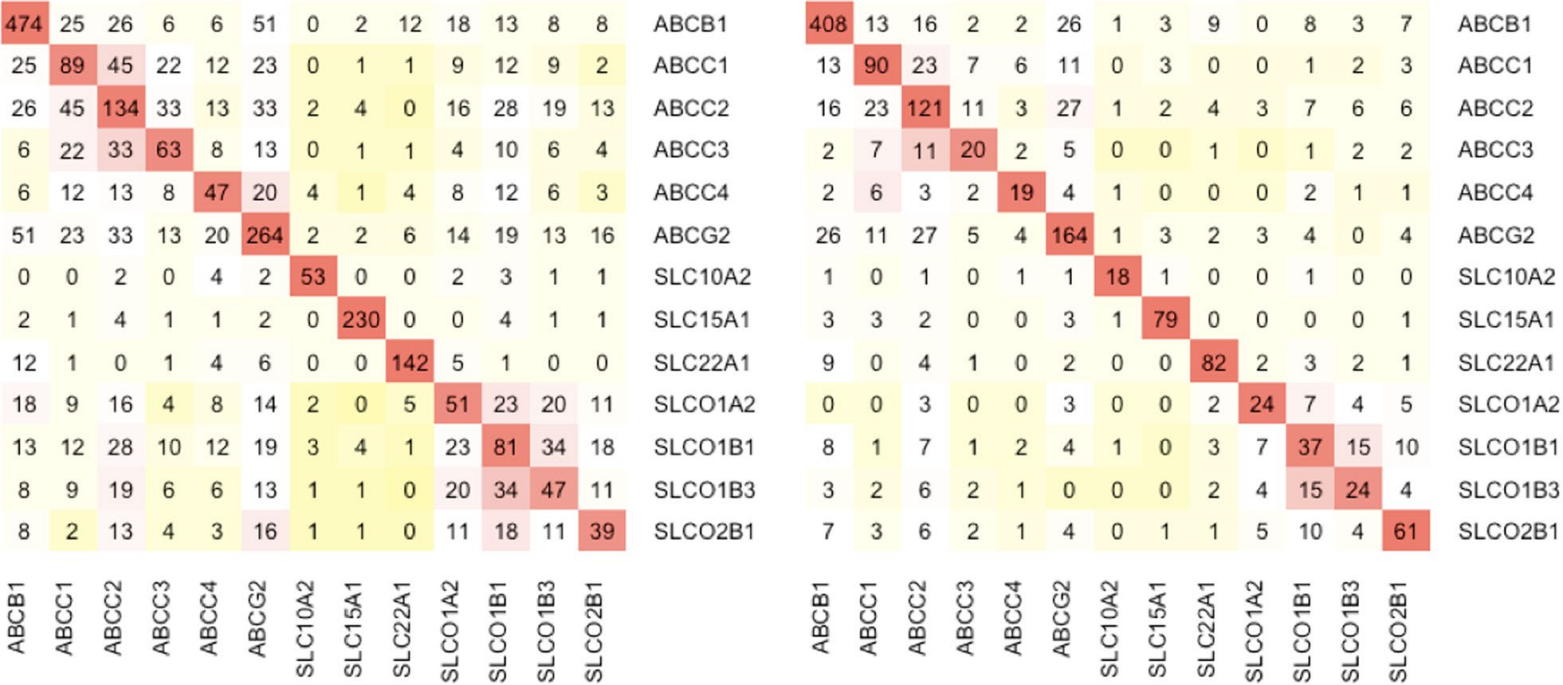
The number of shared substrates and non-substrates between the 13 selected transporters. Transporters can be highly promiscuous with overlapping compound recognition patterns. These two matrices show the number of shared substrates (*left*) and non-substrates (*right*) within the MBTPsubDS1_0a dataset.

The number of substrates (or non-substrates) shared between transporters is shown in Figure 6. It can be seen that in the current dataset there is not enough data to explore the extent to which similarities in transporter sequences determine similarities in compounds transported, since having a more complete data matrix would be required—a selection of compounds tested against a selection of transporters in an all-to-all fashion would be ideal. Furthermore, this dataset contains quite a diverse set of transporters with only OATP1B1 and OATP1B3 sharing a high sequence similarity/identity of 87.1%/78.5% (global pairwise sequence alignment was obtained using EMBOSS Stretcher v6.6.0 [43] with default parameters) followed by the ABCC subfamily (ABCC1 and ABCC3 have sequence similarity/identity of 73.4%/56.7%). We could perhaps perceive from Figure 6 initial support for the assumption that it is more likely that a substrate of SLCO1B1 will also be a substrate of SLCO1B3 than other transporters. However, with many data points missing, this could also be a consequence of experimental assays being carried out more often for transporters belonging to the same family (if more than one transporter was explored in the experiment).

The following two lists summarize the main intended uses of Metrabase (an example is shown in Figure 3):

- read across using similarity to compounds in the database,
- given a protein, show interacting compounds (including those where interaction was tested but not observed),
- given a compound, show interacting proteins (including those where interaction was tested but not observed),
- given a protein, list its tissue expression levels (qualitative only),
- given a tissue, list protein expression levels for this tissue (qualitative only);

and its benefits:

- action type is specified for all Metrabase compounds,
- negative action types (incl. inactive compounds) are included,
- chemical structures are included in the database, along with compound names,
- cell lines are specified for substrate records linked to primary literature references and we aim to expand this for all the ‘activities’ records,
- records are linked to the publications, which the data was extracted from (allowing thus for easier verification and for getting further information) and
- Metrabase is a publicly available and fully accessible resource.

### Use case 1: identification of drug–drug interactions for atorvastatin

Atorvastatin (Lipitor) is a highly prescribed statin for the treatment of high blood cholesterol and for prevention of incidents that are associated with cardiovascular diseases. The mechanism of action of atorvastatin is inhibition of HMG-CoA reductase, and it is also known to be associated with other proteins, among them transporters [44]. In this use case we show how to identify the transporters with which atorvastatin is associated, and cross-reference them with potential inhibitors of these transporters, which could result in high accumulation of the drug and potential toxic drug–drug interactions by increased bioavailability of the drug [45].

#### Method

1. In the “Search by compound” section, run an exact search for “atorvastatin”.
2. The results show that atorvastatin was found to be a substrate of six transporters (MDR1, MCT1, OATP1B1, OATP1B3, OATP1A2, OATP2B1) and also an inhibitor of five transporters (MDR1, BCRP1, OATP1B1, OATP1B3, OATP2B1) with seven references indicating that it is an OATP1B1 substrate.
3. In “Search by protein” select “OATP1B1” and both “inhibitor” and “inducer”.
4. The results reveal a number of potential drug–drug interactions, among them several references to potentially strong inhibitors, such as cyclosporine.

The results of an exact search for cyclosporine (“Search by compound”) reveal a large number of references in support of cyclosporine being an inhibitor of several transporters, including 12 references for OATP1B1 inhibition, suggesting that the dose of atorvastatin should be lowered when co-administered with cyclosporine.

### Use case 2: Exploring potential drug-food interactions with green tea

Green tea is known to contain high amounts of flavan-3-ols, including catechins of which (−)-epigallocatechin-gallate (EGCG) is the most abundant catechin [46]. In this use case we are employing the molecular structure of catechin to search for similar compounds with annotated transporter interactions. If found this might indicate potential adverse nutrient-drug interactions instigated by consumption of green tea coupled together with drug intake.

#### Method

1. In the “Search by compound” section run a similarity search using the SMILES representation of the catechin molecular structure, (c1cc(c(cc1[C@@H]2[C@H](Cc3c(cc(cc3O2)O)O)O)O)O), and Tanimoto coefficient (TC) cut-off of 0.3.
2. The results show 317 hits found in Metrabase that exhibit structural similarity to the catechin chemical structure. The hits include known catechins such as EGCG (TC = 0.823) and epicatechin (TC = 1.0), drugs such as nadolol (TC = 0.511) and troglitazone (TC = 0.457), and other natural products such as phlorizin (TC = 0.457).
3. Metrabase shows that EGCG has been found to be extensively associated with transporters: as a substrate of MRP1/2, OATP1B3 and OATP1A2, as a non-substrate of MDR1, OATP1B1, OATP2B1 and as an inhibitor of several OATPs such as OATP1A2, OATP1B1 and OATP2B1.
4. Nadolol is annotated as a substrate of MDR1 and OATP1A2 and as an inhibitor of BCRP1.
5. This potential interaction suggests that uptake of nadolol may be affected by co-administration with green tea and thus result in low plasma concentration due to the inhibition of its uptake transporter.

This nutrient-drug interaction was highlighted in a recent publication (not included in this version of Metrabase), which reported low intestinal uptake of nadolol by inhibition of OATP1A2-mediated uptake caused by green tea ingestion [47].

### Future development plans

Further data curation, comprehensive data coverage and improvements are planned for subsequent Metrabase releases, as well as inclusion of additional biochemical annotations (especially with respect to sequence variants due to their importance in phenotypic variation, e.g. in drug metabolism). Moreover, we aim to include species other than human, as well as protein expression levels for diseased tissues, as this is highly relevant in the context of drug discovery. Even though the first release of Metrabase includes calculated molecular properties only, the ‘properties.type’ field values of ‘c’ and ‘e’ are proposed to designate calculated and experimental properties respectively, for possible future addition of experimental properties. The additional ‘activities’ fields, although incomplete as indicated in the Activities section above, were included in the first release and are planned to be completed in the subsequent releases. The selection of transporters, which was mainly steered by the guidelines of the International Transporter Consortium [27], is planned to be extended. We first aim to include additional data points for the 13 Metrabase CYPs before extending the protein list to include other Phase I, II and III enzymes involved in xenobiotic metabolism. Further improvements to the web interface can also be expected.

## Conclusions

Metrabase offers structured and freely accessible manually extracted data on interactions between transport and metabolism related proteins and chemical compounds (the first version’s emphasis being on the transport proteins). It provides not only actions and measured activities, but also chemical structural information, tissue expression data and negative action types, which are essential in modeling activity. In particular, by creating accessible data that can be used in e.g. biochemistry, pharmacology and toxicology, we hope diverse research communities will find Metrabase useful and valuable. The availability of the raw data in an easily accessible form will allow other research workers in the field to easily include this data in collections of transporter/metabolism datasets as well as allow easy integration with other programs and services.

### Availability and requirements

Metrabase is accessible via an online user interface at http://www-metrabase.ch.cam.ac.uk. Users can also install their own local copy of the database, for which a MySQL server installation is required. The Download section provides access to a MySQL dump of Metrabase, its schema, a user manual providing comprehensive information and versions of the transporter substrate dataset, i.e. MBTPsubDS. Metrabase version 1.0 is freely available under a Creative Commons Attribution-ShareAlike 4.0 International license. However, the integrated data retains the licensing of the original data sources. The TP-Search and ChEMBL records may have been modified and augmented based on literature revision or reinterpretation, while the HPA records were included unmodified. The ‘datasource id’ and ‘datasource version’ fields indicate the source of each relevant Metrabase record.

## Additional files

Additional file 1: The transporter substrate dataset. The Additional file 1 contains data for compounds that were found to be (or nor to be) substrates of transporters (all records involving the conflicting action types were excluded; MBTPsubDS1_0.csv). Additional file 2: The transporter substrate dataset. The Additional file 2 contains data for compounds that were found to be (or nor to be) substrates of transporters (MBTPsubDS1_0a.csv).

## Abbreviations

ABC: ATP-binding cassette
ADME: absorption, distribution, metabolism and excretion
ATP: adenosine triphosphate
CSV: comma-separated values
CYP: cytochrome P450
EGCG: (–)-epigallocatechin-gallate
EMA: European Medicines Agency
FDA: US Food and Drug Administration
HGNC: HUGO Gene Nomenclature Committee
HMG-CoA: 3-hydroxy-3-methylglutaryl-coenzyme A
HPA: Human Protein Atlas
InChI: IUPAC International Chemical Identifier
MBTPsubDS: Metrabase transporter substrate dataset
(Q)SAR: (quantitative) structure–activity relationship
SLC: solute carrier
SQL: structured query language
TSV: tab-separated values
